# Inhibition of galectins in cancer: Biological challenges for their clinical application

**DOI:** 10.3389/fimmu.2022.1104625

**Published:** 2023-01-10

**Authors:** Diego José Laderach, Daniel Compagno

**Affiliations:** ^1^Molecular and Functional Glyco-Oncology Laboratory, Instituto de Química Biológica de la Facutad de Ciencias Exactas y Naturales (IQUIBICEN-CONICET), Buenos Aires, Argentina; ^2^Departamento de Química Biológica, Facultad de Ciencias Exactas y Naturales, Universidad de Buenos Aires, Buenos Aires, Argentina; ^3^Departamento de Ciencias Básicas, Universidad Nacional de Luján, Luján, Argentina

**Keywords:** galectins, galectin inhibitors, cancer treatments, tumor microenvironment, medical intervention for cancer

## Abstract

Galectins play relevant roles in tumor development, progression and metastasis. Accordingly, galectins are certainly enticing targets for medical intervention in cancer. To date, however, clinical trials based on galectin inhibitors reported inconclusive results. This review summarizes the galectin inhibitors currently being evaluated and discusses some of the biological challenges that need to be addressed to improve these strategies for the benefit of cancer patients.

## Introduction

Galectins are a family of proteins defined by their Carbohydrate Recognition Domain (CRD). Through that domain, galectins bind to galactosides, such as N-acetyllactosamine residues attached to biomolecules (1). Interestingly, the binding of glycans to galectins’ CRD is subject to allosteric regulations (2, 3). Even if carbohydrate binding is the classifying criteria for these proteins, it has long been known that galectins can also interact with other biological molecules in a carbohydrate-independent manner (4) [reviewed in (5, 6)]. Altogether, the list of galectin interactors reported so far has dramatically grown in the last years (extensive bibliography (7–12), cited as examples). Through this panoply of interactions, galectins regulate physiological cell properties such as differentiation; adhesion and migration; cell cycle and survival, immune patrolling, RNA splicing, and gene transcription (5, 6, 13).

Expression of galectins is strongly altered in cancer; comprehensive reviews address this point elsewhere (8, 14, 15). Albeit not oncogenic drivers, galectins exacerbate the malignant phenotype (16–18). Indeed, galectins regulate homotypic and heterotypic aggregation of cancer cells, cancer cell migration and invasion [reviewed in (17)], tumor angiogenesis [reviewed in (19, 20)] and immune escape [reviewed in (7, 8, 15)]. Consequently, increased galectin production in cancers generally predicts a poor clinical outcome for patients (21–24). Among the 16 galectins identified in mammals (12 in humans, as found in GenBank https://www.ncbi.nlm.nih.gov/genbank/ accessed on 20 November 2022), galectins-1, -3, -7, -8, and -9 have been extensively evaluated in cancer patient samples. Pre-clinical experimentation has demonstrated that galectin inhibitors are interesting anti-tumor tools, particularly when combined with irradiation (25–34), chemo- (34–42), anti-angiogenic- (43, 44), and immune-therapies (37, 45, 46). Interestingly, some of the described galectin inhibitors are currently being evaluated at the clinical level. This review aims to summarize galectins’ inhibitory strategies being tested, those that gave encouraging results in pre-clinical studies, and the challenges their effective use may entail.

## Current galectin inhibitors

**Figure 1:**
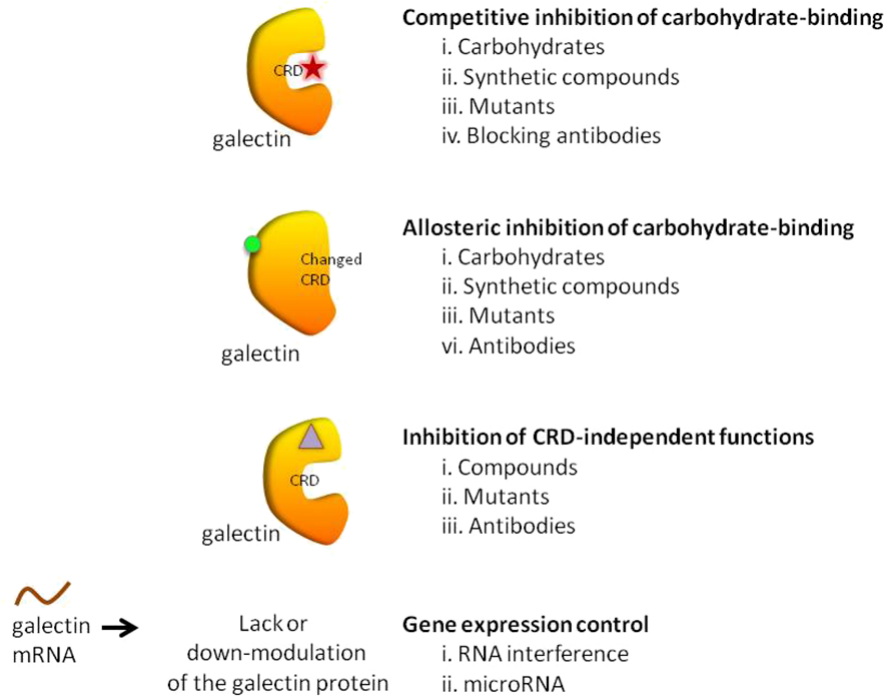
Galectin inhibitory strategies.

Current galectin inhibitors are listed in **Table 1** (*in vivo* pre-clinical evaluations) and **Table 2** (clinical trials). This topic was previously covered by (112–116). However, this manuscript aims to update on the current developments in the field, including some strategies not previously considered. It also assesses the challenges to scaling up the use of galectin inhibitors in the clinic. In this review, compounds are classified according to their mechanism of action (their influence over CRD -competitive vs. allosteric inhibitions-) or their glycan independence (**Figure 1**).

**Table 1:** *In vivo* pre-clinical studies with galectin inhibitors.

Inhibitor	Structure	Pre-clinical model	References
a) Carbohydrate compounds			
β-D-lactosyl-steroid	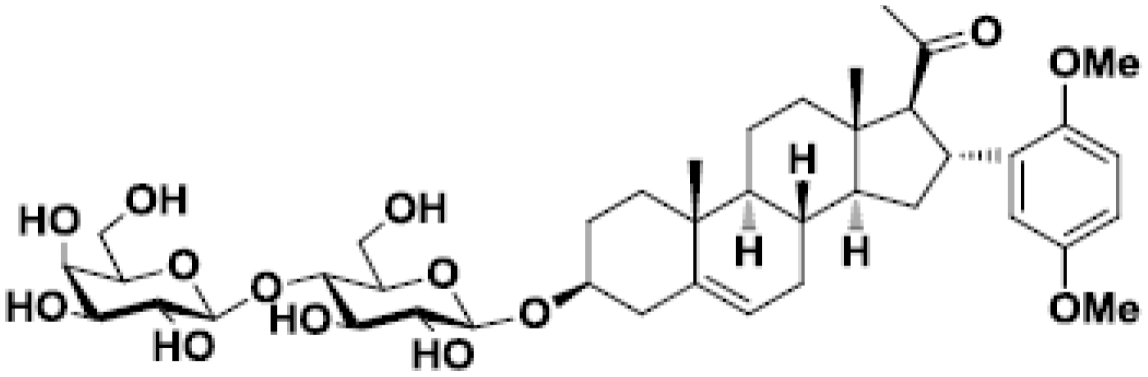	Lymphoma and glioblastoma	(47)
Thiodigalactose (TDG)	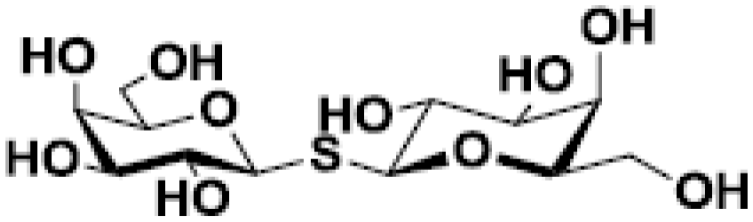	Pulmonary metastasis in murine breast and colon cancer models	(48, 49)
Modified-thiodigalactose (TD139)	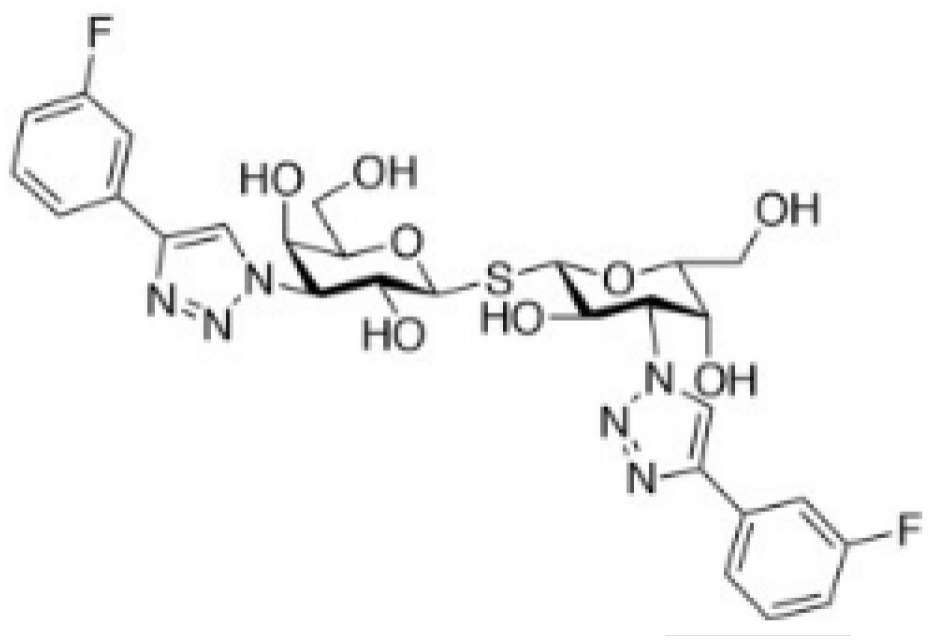	Lung fibrosis	(50, 51)
GB1107	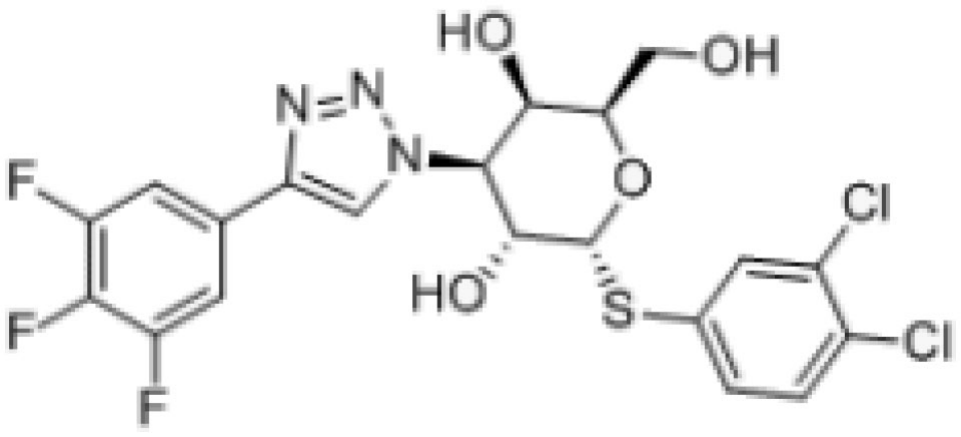	Human and mouse lung adenocarcinoma in murine models Synergy with negative immune checkpoint. Oral squamous cell carcinoma; synergy with cetuximab	(52, 53) (54)
Lactulose-L-leucine	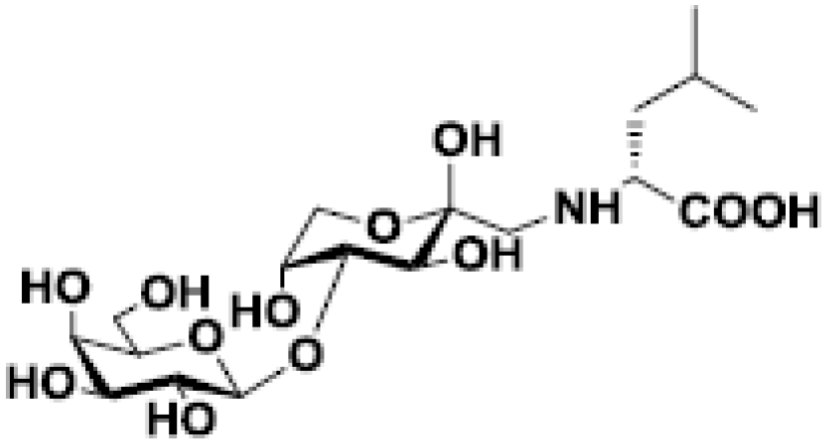	Breast and prostate cancers in murine models	(55, 56)
Dendrimers : galactose- or lactose-conjugated porphyrin derivatives	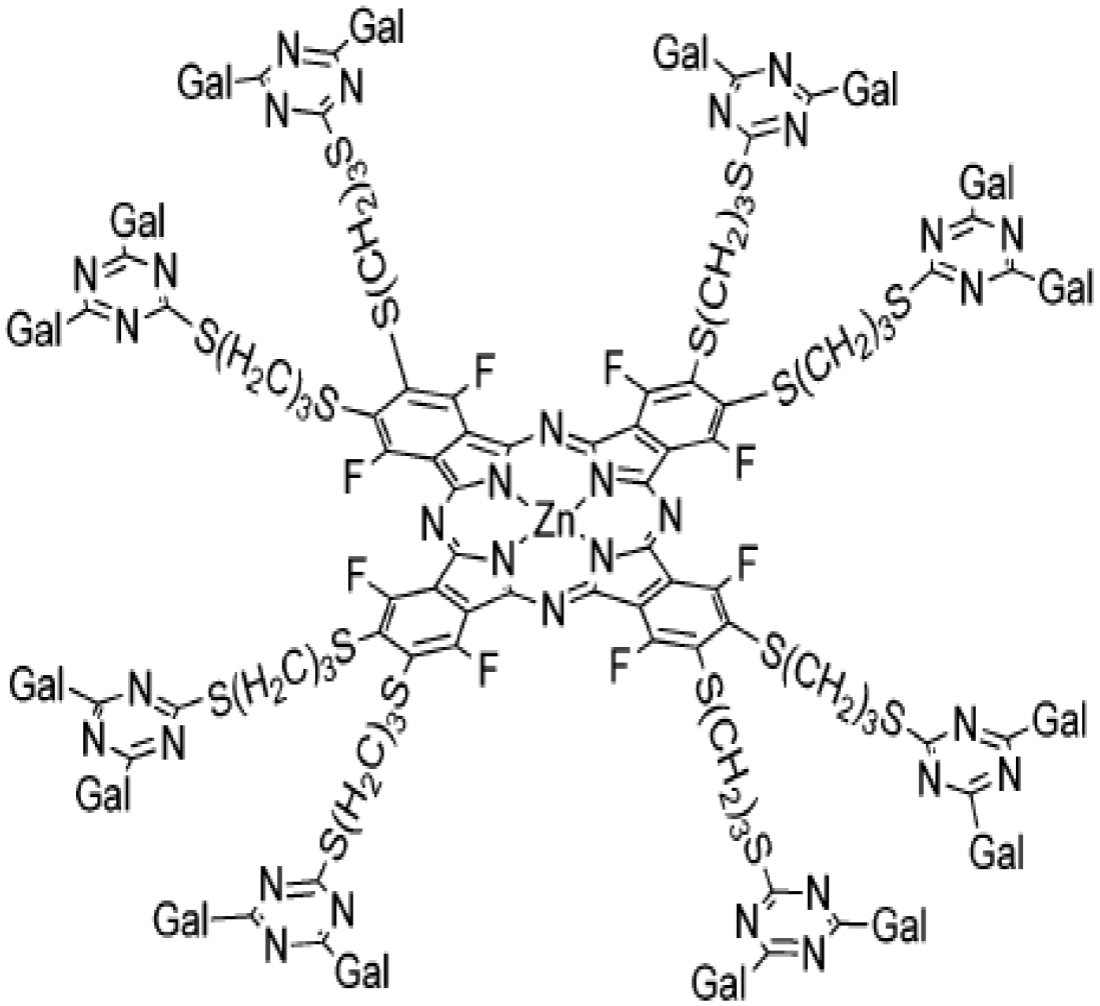	Photodynamic anti-tumor therapy Bladder cancer model Radiation-induced fibrosarcoma	(57) (58)
Modified citrus pectin (MCP)	Heterogenous chemical definition, with the following general structure 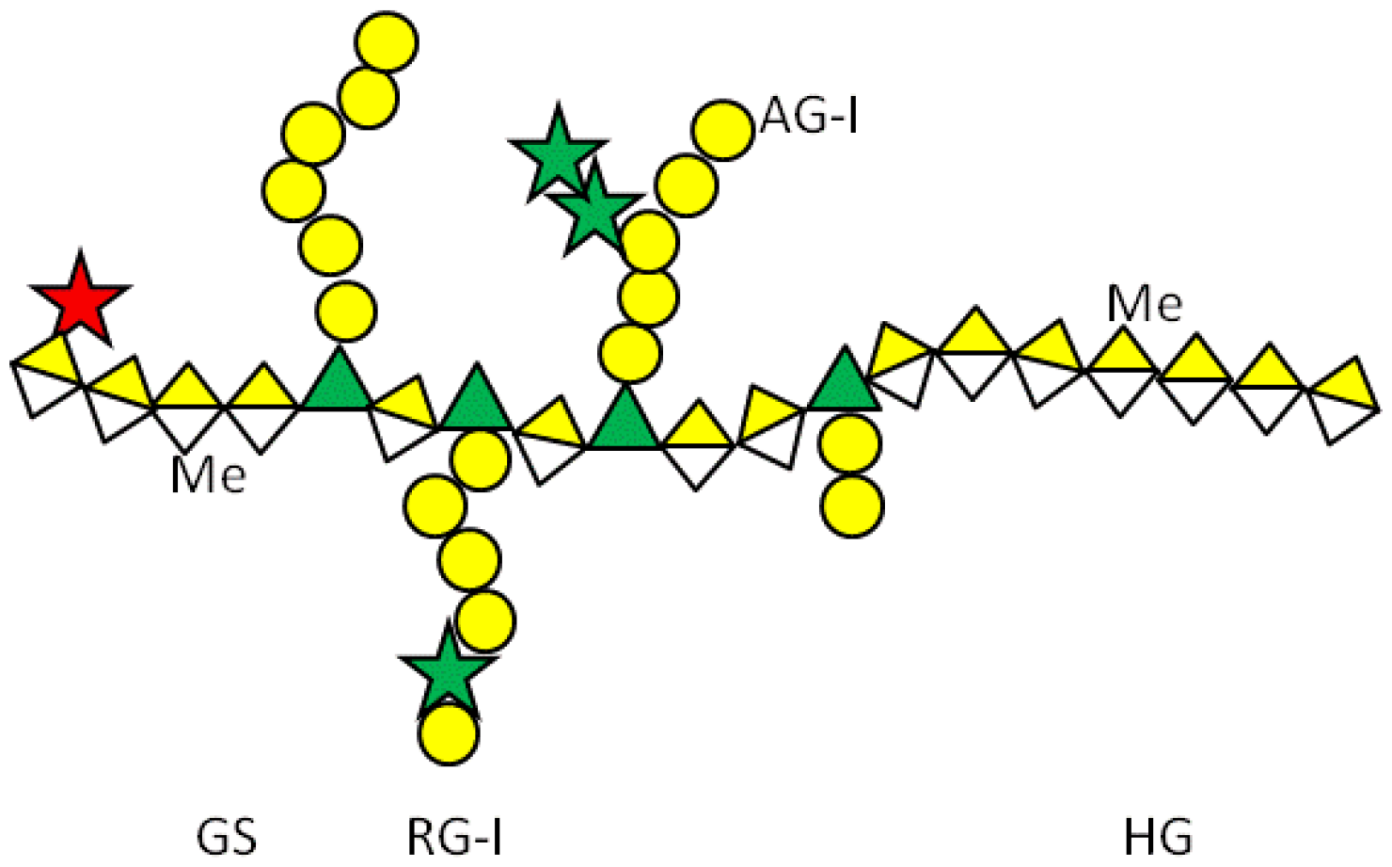 Several methods of preparation: US7491708B1, ES2537936B1, US 2016/0030467 A1 patents	Melanoma Thyroid cancer Breast and colon cancers Prostate cancer	(59) (60) (61) (62)
PectaSol-C	Derived from MCP Low molecular weight, 5 % galacturonic acid US 2011/0294755A1 patent, EcoNugenics	Not *in vivo* pre-clinical studies in animals found (only original MCP)	
GCS-100	derived from MCP US8877263B2 patent, La Jolla Pharmaceutical Company	Mastocytoma	(63)
GM-CT-01 or DAVANAT	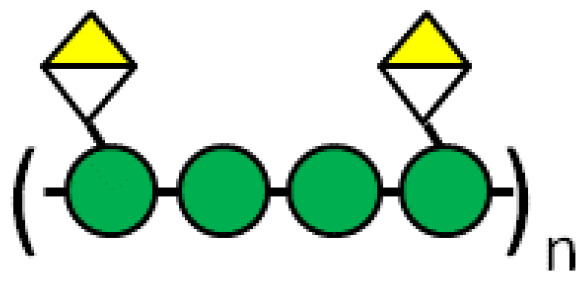 US 2014/0235571 A1 patent, Galectin Therapeutics Inc	Toxicity studies on mice, rats and dogs Colon Cancer	(64) (64)
GR-MD-02 (belapectin)	1,4-linked (methyl) galacturonic acid backbone interspersed with α-1,2 linked rhamnose, the rhamnose carrying 1,4-β-D-galactose residues or 1,5-α-L-arabinose oligomers. 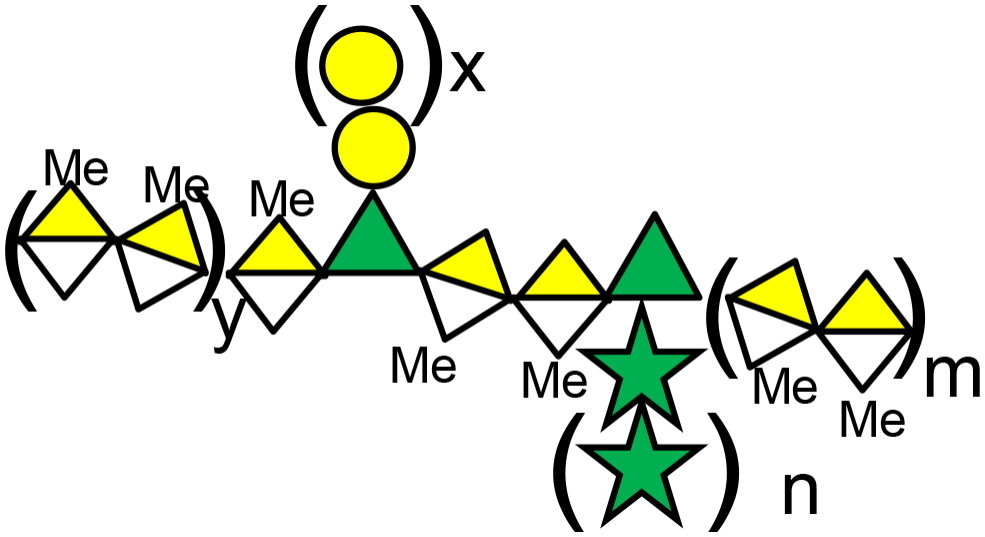 US8871925B2 patent, Galectin Therapeutics Inc.	Sarcoma, breast, and prostate cancer	(65)
Carbohydrate-complexed nanoparticles	Citrus pectin-nanoparticles Galactose-Tuftsin peptide-nanoparticles	Colon cancer Melanoma	(66) (67)
b) Peptides, peptidomimetics and proteins			
Anginex peptide	ANIKLSVQMKLFKRHLKWKIIVKLNDGRELSLD	*In vivo* angiogenesis	(68)
		Teratocarcinoma	(69)
		Melanoma, Ovarian and breast carcinoma	(26, 32, 43, 44)
Peptidomimetics: 6DBF7 dibenzofuran (DBF)-modified peptide	[DBF] 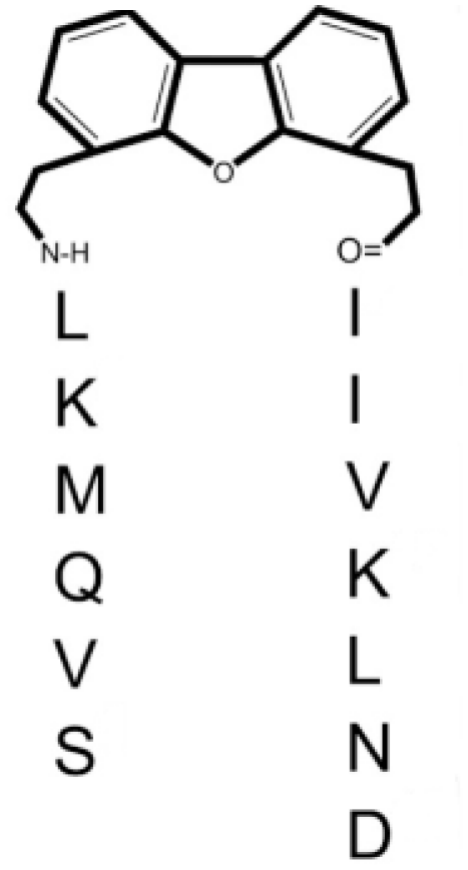	Melanoma, lung, and ovarian carcinoma	(70, 71)
DB16	SVQMKL-[DBF]-AIVKLNA	Melanoma, lung, and ovarian carcinoma	(71)
DB21	SVQNvaKL-[DBF]-IIVKLNA	Melanoma, lung, and ovarian carcinoma	(71)
OTX008	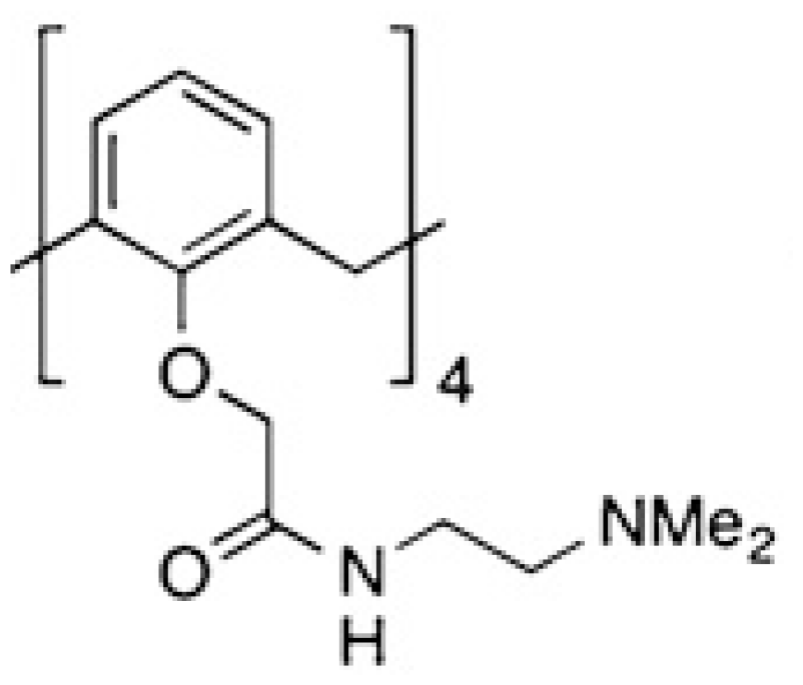	Melanoma, glioblastoma, thyroid and ovarian carcinoma	(40, 43, 72, 73)
PTX013	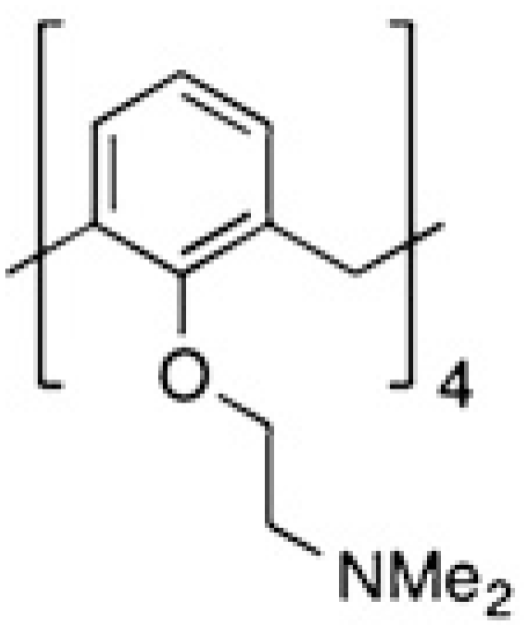	Melanoma	(74)
Dominant negative mutants	Gal-3C (lacks N terminal)	Multiple myeloma	(75)
		Breast cancer	(76)
		Ameliorates heart failure after myocardial infarction	(77)
	Gal-3 (Ser6-->Glu Ser6-->Ala) mutant unable to phosphorylate	Breast cancer	(78)
Neutralizing antibodies	anti-galectin-1-mAb	Head and neck cancer	(45)
		Lung carcinoma and melanoma	(79)
		Kaposi' s sarcoma	(80)
	anti-galectin-3-mAb	Breast and ovarian cancers	(81)
	anti-galectin-9 mAb	Colon adenocarcinoma	(82)
		Breast cancer	(83)
		Pancreatic carcinoma	(84)
		Myeloid Leukemia	(85)
c) Oligonucleotides			
Aptamers	AP-74 M-545 DNA aptamer (galectin-1 specific)	Lung cancer	(86)
siRNA and shRNA-coding vectors (few exemples cited)	galectin-1 shRNA	Hepatocellular carcinoma	(87)
		Peripheral nerve sheath tumors	(88)
		Gastric cancer	(89)
		Osteosarcoma	(90)
		Lung carcinoma	(91)
		Glioblastoma	(37, 92–95)
		Prostate cancer	(96)
		Melanoma	(97, 98)
		Kaposi's sarcoma	(80)
	galectin-3 shRNA	Hepatocellular carcinoma	(99)
		Melanoma	(100)
		Pancreatic cancer	(101)
		Prostate cancer	(102)
	galectin-8 shRNA	Prostate cancer	(103)
	galectin-4 shRNA	Colorectal cancer	(104)
Regulation of mi-RNA	miR-424-3p (galectin-3) using resveratrol	Ovarian and colorectal cancers	(105)
d) Compounds from chemical synthesis			
Benzimidazole compounds	LLS30 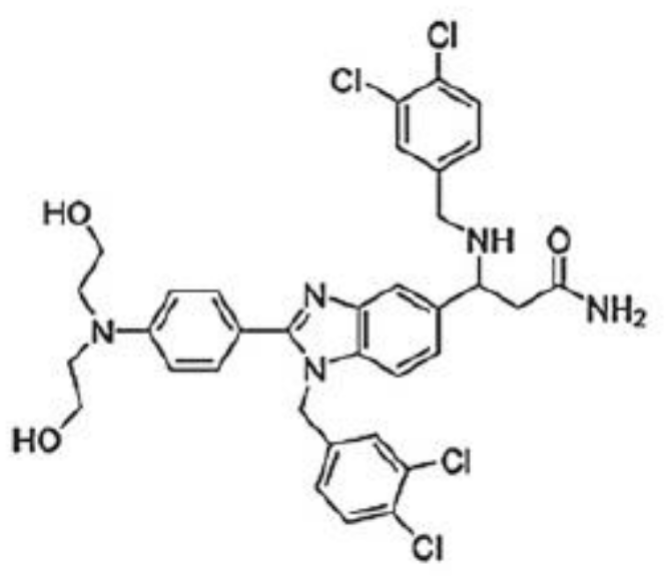	Ovarian cancer Prostate cancer	(106) (107)
	LLS2 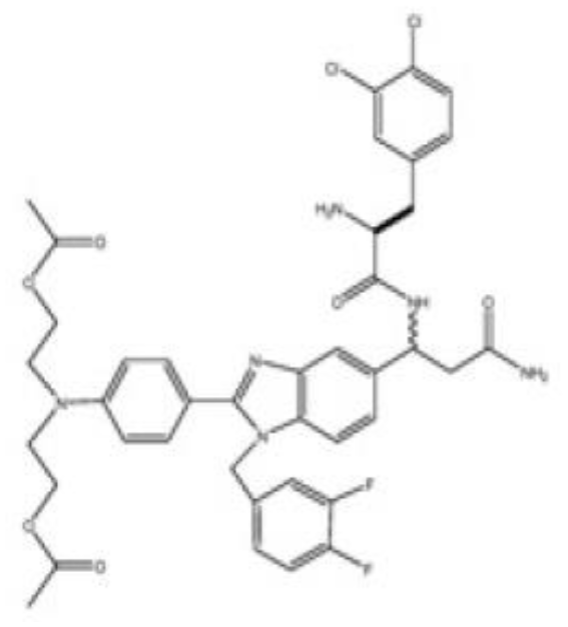	Peripheral nerve sheath tumors	(88)

Glycans symbols (according to https://www.ncbi.nlm.nih.gov/glycans/snfg.html).

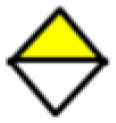
D-galacturonic acid, 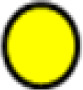 D-galactose, 
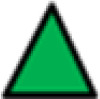
 L-rhamnose, 
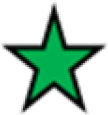
 L-arabinose, 
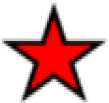
 D-xylose, Me: methyl ester

**Table 2:** Clinical trials with galectin inhibitors.

Clinical trial #	Phase	Inhibitor	Combinatory treatment	Targeted-galectin (reported)	Disease	Last Update	Status (mention if the results are available)
Healthy subjects							
NCT03809052	I	GB1211	None	gal-3	Healthy subjects	March 17, 2021	Completed, with results
Cancers							
NCT05240131	I/II	GB1211	Atezolizumab	gal-3	Non-small cell lung cancer	October 3, 2022	Recruiting
NCT01681823	II	PectaSol-C	None	gal-1/-3	Biochemical relapsed prostate cancer	January 29, 2020	Completed (108, 109),
NCT00514696	II	GCS-100	None	gal-3	Chronic lymphocytic leukemia	June 17, 2013	Completed, unreported results
NCT00776802	I/II	GCS-100	Etoposide/Dexamethasone	gal-3	Relapsed/refractory diffuse large B-cell lymphoma	June 25, 2013	Withdrawn (Lack of funding), unreported results
NCT00609817	I	GCS-100	Bortezomib/Dexamethasone	gal-3	Relapsed/refractory multiple myelome	June 25, 2013	Terminated (Lack of funding), unreported results
NCT00054977	I	GM-CT-01	5-Fluorouracil	gal-1/-3	Advanced solid cancers: colorectal, lung, head and neck, and prostate cancers	March 12, 2012	Completed, unreported results
NCT00388700	II	GM-CT-01	5-Fluorouracil, Leucovorin, bevacizumab	gal-1/-3	Colorectal cancer	February 14, 2018	Withdrawn (Financing and re-organization), unreported results
NCT00110721	II	GM-CT-01	5-Fluorouracil	gal-1/-3	Colorectal cancer	March 6, 2012	Terminated (study protocol amended to a new treatment regimen: study DAVFU-006.), unreported results
NCT00386516	II	GM-CT-01	5-Fluorouracil	gal-1/-3	Advanced gall bladder and bile duct cancer	August 1, 2017	Withdrawn (Financing and re-organization), unreported results
NCT01723813	I/II	GM-CT-01	Peptide vaccination	gal-1/-3	Metastatic melanoma	March 12, 2019	Terminated due to end of validity of the peptide vaccine; no reported results, unreported results
NCT02117362	I	GR-MD-02	Ipilimumab	gal-1/-3	Metastatic melanoma	March 21, 2019	Completed, unreported results
NCT00054977	I	GR-MD-02	5-fluorouracil	gal-1/-3	Advanced solid tumors: colorectal, lung, breast, head and neck, prostate	March 12, 2012	Completed, unreported results
NCT02575404	I	GR-MD-02	Pembrolizumab	gal-1/-3	Advanced melanoma, non-small cell lung cancer, and head and neck squamous cell cancer	July 15, 2022	Active, not recruiting (110)
NCT04987996	II	GR-MD-02	Pembrolizumab	gal-1/-3	Metastatic melanoma, head and neck squamous cell carcinoma	September 10, 2022	Suspended (Study delayed due to ongoing discussions with the owner of one of the investigational agents), unreported results
NCT02117362	I	GR-MD-02	Ipilimumab	gal-1/-3	Metastatic melanoma	March 21, 2019	Completed, unreported results
NCT01724320	I	OTX008	None	gal-1	Advanced solid tumors	November 9, 2012	Unknown, unreported results
NCT04666688	I/II	Lyt-200	Chemotherapy, Anti-PD-1	gal-9	Relapsed/refractory metastatic solid tumors	March 11, 2022	Recruiting
Non-cancer diseases							
NCT02257177	I/II	TD139		gal-3/others	Idiopathic pulmonary fibrosis	April 8, 2021	Completed, with results
NCT03832946	II	TD139		gal-3/others	Idiopathic pulmonary fibrosis	May 24, 2022	Active, not recruiting
NCT04473053	I/II	TD139		gal-3/others	COVID-19	September 16, 2021	Active, not recruiting
NCT04607655	I/II	GB1211		gal-3	Non-alcoholic steatohepatitis (NASH) and liver fibrosis	February 4, 2021	Withdrawn (Due to COVID-19 pandemic and change in the clinical development strategy for the GB1211 compound), unreported results
NCT05009680	I/II	GB1211		gal-3	Hepatic impairment	August 3, 2022	Active, not recruiting
NCT01960946	I/II	MCP/PectaSol C		gal-1/-3	Hypertension	February 21, 2021	Completed, results in (111)
NCT01717248	I	GCS-100		gal-3	Chronic kidney disease	June 20, 2013	Completed, unreported results
NCT01843790	II	GCS-100		gal-3	Chronic kidney disease	September 1, 2015	Completed, unreported results
NCT02312050	II	GCS-100		gal-3	Chronic kidney disease	May 19, 2015	Unknown, unreported results
NCT02155673	II	GCS-100		gal-3	Chronic kidney disease	December 26, 2016	Completed, unreported results
NCT02333955	II	GCS-100		gal-3	Chronic kidney disease	January 15, 2015	Withdrawn (Corporate decision), unreported results
NCT01899859	I	GR-MD-02		gal-1/-3	Non-alcoholic steatohepatitis, portal hypertension, and advanced liver fibrosis	February 23, 2015	Completed, unreported results
NCT02462967	II	GR-MD-02		gal-1/-3	Portal hypertension, and advanced liver fibrosis	October 8, 2020	Completed, with results
NCT02421094	II	GR-MD-02		gal-1/-3	Liver fibrosis	October 8, 2020	Completed, with results
NCT02407041	II	GR-MD-02		gal-1/-3	Psoriasis	September 7, 2020	Completed, with results
NCT04332432	I	GR-MD-02		gal-1/-3	Subjects with normal hepatic function and subjects with hepatic impairment	March 28, 2022	Completed, unreported results
NCT04365868	IIb/III	GR-MD-02		gal-1/-3	Esophageal varices in NASH cirrhosis	September 22, 2022	Recruiting

Data from www.clinicaltrials.gov. [Accessed November 24, 2022].

### Galectin inhibitors affecting carbohydrate recognition

#### Competitive inhibitors of carbohydrate-binding to galectins

The lectin functions of this family of proteins are the most widely studied. Indeed, galectins bind to β-galactosides through their CRD. For instance, considering its canonical ligand lactose, the C4’ and C6’ hydroxyls of the galactose and C2 and C3 of glucose are primarily responsible for the hydrogen-bond interactions with conserved residues of CRD in galectin-3 (117) and galectin-1 (118). Basis of the molecular glycan-protein interactions has also been described for other galectins (119, 120). The fine specificity of galectins for different oligosaccharides stems from residues surrounding this main binding site. Consequently, each galectin has a different glycan-binding preference contributing to its specific biological activities (121). The first described galectin inhibitors are molecules capable of binding to the CRD and preventing further ligand binding. Galectin inhibitors based on these competitive interactions consist of chemically modified mono or disaccharides structured around galactose (58, 122–125), lactose (58, 125–127), thiodigalactose (TDG) (34, 128–132), talose (133, 134) and lactulose (135). One of the first tempts to use this type of inhibitor in cancer consisted of administering a β-D-lactosyl-steroid. This treatment significantly increased the survival of mice grafted with lymphoma and glioblastoma cells (47, 136). Moreover, this compound increases the anti-tumor cytotoxic effects of cisplatin in mice (47).

Several chemical modifications of glycans have been developed to improve these molecules’ inhibitory properties. For example, introducing a sulfur atom into the glycoside linkage in TDG makes the molecule more resistant to glycosidases (137). The *in vivo* anti-tumor properties of some of these compounds were challenged in pre-clinical studies. For instance, TDG administration reduces pulmonary metastasis in murine breast and colon cancer models (48). TDG promotes immune infiltration, reduces angiogenesis, and protects cells against oxidative stress (49). The most advanced TDG in clinical studies is TD139 (also named as GB0139), developed by Galecto Biotech (Copenhagen, Denmark). TD139 recognizes galectin-3 CDR with high affinity (Kd 68 nM) (138). However, its absolute selectivity for galectin-3 is relative since it also binds to galectin-1 CDR (Kd 220 nM) and other galectins with lower affinities (138). This compound was initially evaluated in pre-clinical models of lung fibrosis (50, 51). Interestingly, TD139 was also evaluated in a clinical trial as a potential therapeutic for idiopathic pulmonary fibrosis (NCT02257177; www.clinicaltrials.gov [accessed November 24, 2022]; **Table 2**) (139).

More recently, a series of monosaccharide galectin-3 inhibitors with high affinities and good selectivity over other galectins have been described (140). From this series, GB1107 (3,4-dichlorophenyl 3-deoxy-3-[4(3,4,5-trifluorophenyl)-1H-1,2,3- triazol-1-yl]-1-thio-α-D-galactopyranoside) from Galecto Biotech; has good affinity (Kd 37 nM) and bind to the CRD of galectin-3. Both, TD139 and GB1107 are membrane-permeable small molecules (141). GB1107 is characterized by good biodisponibility upon oral administration and low clearance (52). It was demonstrated that the oral administration of GB1107 reduced human and mouse lung adenocarcinoma growth and blocked metastasis in murine models (52). Mechanistically, treatment with GB1107 promotes tumor M1 macrophage polarization and CD8(+) T-cell infiltration (52). Moreover, GB1107 potentiated the effects of a PD-L1 immune checkpoint inhibitor to increase expression of cytotoxic (IFNgamma, granzyme B, perforin-1, Fas ligand) and apoptotic (cleaved caspase-3) effector molecules (52, 53). In addition, GB1107 and cetuximab displayed a synergistic inhibitory effect on the growth of oral squamous cell carcinoma (54). Phase I studies with GB1211 (which shares a chemical template with GB1107) have been completed (NCT03809052, **Table 2**), and Galecto Biotech initiated safety and efficacy clinical studies with GB1211 combined with atezolizumab in the treatment of non-small-cell lung cancer (NCT05240131, **Table 2**).

Finally, it should be mentioned that chemical modifications of galactosides and their evaluation as galectin inhibitors in cancer are an intense field of research. First, synthetic glycoamines evidenced anti-tumor activity (55, 56, 142, 143). Indeed, lactulose-L-leucine mimics cancer-associated Thomsen-Friedenreich glycoantigen and binds to galectin-3. At a molecular level, it was demonstrated that this compound binds to the CRD of galectins-1 and -3 with higher affinity than lactose and TDG (135). In a murine breast cancer model, the administration of lactulose-L-leucine (and fructosyl-D-leucine) inhibited spontaneous metastasis in nude mice (56). The same group demonstrated the beneficial effects of lactulose-L-leucine in controlling and preventing prostate cancer metastasis to the bone (55). Other inhibitory molecules arising from chemical modifications of galactosides can also be cited (122, 144–146); however, they do not reach the level of *in vivo* evaluation.

To improve galectin inhibitors’ properties, inspiration was found in the clustering nature of galectin glycan interactions. Indeed, the synthesis of multivalent glyco-clusters with improved galectin inhibitory potential has been reported (147–152). Interestingly, cell aggregation can either be inhibited or enhanced depending on the number of lactose groups in functionalized dendrimers (153). Unfortunately, no evaluation of their *in vivo* biological effects in pre-clinical models was yet reported. Another strategy based on the same conceptual framework tested dendrimers obtained by galactose conjugation to the porphyrin derivatives (154). In this case, a photodynamic anti-tumor therapy was successfully reported in a pre-clinical *in vivo* bladder cancer model (57).

Pectins are another group of galectin-binding, inhibitory compounds. Natural pectins are large and heterogeneous polysaccharides found in plants which constitutes fiber components of our diet. Pectins have molecular weights ranging from 60 to 130 kDa and are constituted by three main polysaccharides: homogalacturonan (HG), rhamnogalacturonan-I (RG-I), and substituted galacturonans (GS) (155, 156). Pectins must be modified by pH and heat to gain solubility and biological effects. Indeed, hydrolysis induces galactoside exposure, and now, modified pectins bind galectins (157, 158). Contrary to what was often supposed, some experimental data prompt the existence of non-conventional sites of pectin binding in galectins (159–162). On the contrary, other *in vitro* data support that pectin-mediated biological effects are (or partially are) mediated by glycans (163–166). Adding complexity to the field, modified pectins are generally administered orally. Nevertheless, pectins are not digestible in the human intestinal tract, and their modifications are believed to increase their absorbability (167, 168). Moreover, it has been postulated that products of pectin fermentation by the human microbiota should contribute to their systemic *in vivo* biological effects (169). It should also be mentioned that pectins induce galectin-independent biological effects (170, 171). Altogether, these arguments indicate that more basic research is needed to clarify the fine mechanisms through which pectins induce their biological effects.

In this context, one of the most studied galectin inhibitors is the modified citrus pectin (MCP), which is obtained by partial hydrolysis of citrus pectin. *In vitro* studies demonstrated that MCP binds galectin-3 through galactoside residues (59, 62). Functionally, MCP inhibits galectin-3 binding to endothelial cells, and more importantly, the adhesion of breast tumors to endothelial cells (61). In addition, MCP treatment induces important metabolic changes in tumor-associated macrophages, which impacts on tumor growth and metastasis (172, 173). Interestingly, these MCP biological effects are carbohydrate dependent (59). *In vivo* administration of MCP inhibits melanoma (59), thyroid (60), breast and colon tumor growth, angiogenesis and metastasis (61, 173), and spontaneous metastasis in a rat prostate cancer model (62). Due to the high chemical variability of dietary MCP supplements on the market, more defined MCP variants have been described: PectaSol-C, GCS-100, GM-CT-01 and GR-MD-02. PectaSol-C has a molecular weight ranging from 5-10 kDa with 5% of monogalacturonic acid content (174). *In vitro* studies demonstrated the potential interest of PectaSol-C MCP in prostate (174, 175), breast (175) and ovarian cancers (176, 177), particularly if used combined with other therapies (175, 177). Interestingly, phase II pilot studies demonstrated the tolerability and encouraging biological results obtained by the use of this inhibitor in prostate patients (108, 109) (NCT01681823, **Table 2**).

GCS-100 is a complex polysaccharide prepared from modified citrus pectin. Mechanistically, GCS-100 detaches galectin-3 from CD4+ and CD8+ tumor-infiltrating lymphocytes, boosts cytotoxicity and restores IFN-gamma secretion (63). Similar effects were obtained by using N-acetyllactosamine, suggesting GCS-100 effects are carbohydrate-dependent (63). Interestingly, GCS-100 induces tumor rejection only when associated with vaccination in pre-clinical model of mastocytoma secretion (63), implying GCS-100 modulates the tumor immune attack. Altogether, these promising results prompt La Jolla Pharmaceuticals to launch GCS-100-based clinical trials. Following a phase I dose escalation safety study in patients with refractory solid tumors (178), a phase II study was completed in patients with chronic lymphocytic leukemia (179) (NCT00514696, **Table 2**). In these exploratory trials, GCS-100 was well tolerated, and 25% of patients showed a partial response (179). In addition, the use of GCS-100 has also been evaluated in chronic kidney disease (Phase I NCT01717248 and phase IIa NCT01843790, **Table 2**). In 2015, La Jolla Pharmaceuticals announced that they were discontinuing the development of GCS-100 after the Food and Drug Administration (FDA) required a more complex characterization of the compound to advance into late-stage development (NCT00776802 and NCT00609817, **Table 2**).

Another pectin-derived polysaccharide able to inhibit galectins is GM-CT-01 or DAVANAT^®^. This polysaccharide is extracted from guar seeds and subjected to controlled partial chemical degradation (developed by Galectin Therapeutics, formerly Pro-Pharmaceuticals). A backbone of the galactomannan is composed of (1→4)-linked β-D-mannopyranosyl units, to which single α-D-galactopyranosyl is attached by (1→6)-linkage (64). The average repeating unit of GM-CT-01 consists of seventeen β-D-Man residues and ten α-D-Gal residues (Man/Gal ratio is 1.7), and an average polymeric molecule contains approximately 12 of such repeating units (for the average molecular weight of 51,000 Da). *In vitro*, GM-CT-01 boosts the cytotoxic properties of CD8(+) tumor-infiltrating lymphocytes and their ability to produce IFN-gamma (180). Indeed, this pectin prevents glycosylated cytokines (IFNγ between others) be captured by galectin-3 and therefore allowing the chemokine gradient needed to attract lymphocytes towards the tumor (181). Pre-clinical studies in mice defined GM-CT-01 non-toxic doses (alone or combined with other chemotherapies) (64). Moreover, such studies demonstrated GM-CT-01 beneficial effects in colon cancer models (64). Interestingly, a phase I clinical trial was completed in cancer patients with advanced solid tumors by administration of DAVANAT^®^ combined with 5-fluorouracil treatment (NCT00054977, **Table 2**). Combinatory treatment was well-tolerated. While phase II trials were announced, these trials were never initiated, having a “withdrawn/terminated status” in www.clinicaltrials.gov (NCT00388700, NCT00110721, NCT00386516, **Table 2**). In addition, melanoma peptide vaccination plus GM-CT-01 was evaluated in melanoma (NCT01723813). This clinical trial was “terminated due to end of validity of peptide vaccine” with no reported results.

Finally, GR-MD-02 (belapectin) is a galactoarabino-rhamnogalacturonan-rich polysaccharide obtained through chemical processing from apple pectin (developed by Galectin Therapeutics, Norcross, Georgia, USA). GR-MD-02 is a galectin-3 inhibitor which synergizes with anti-OX40 treatment to promote tumor regression and increases survival of tumor-bearing mice (65). This occurs through a CD8(+) T cell-dependent mechanism, reducing the immunosuppresion mediated by myeloid-derived suppressor and regulatory Foxp3(+)CD4(+)T cells (65). GR-MD-02 administration induced a significant reduction of liver fibrosis in experimental models of non-alcoholic steatohepatitis (182, 183). GR-MD-02 is being evaluated in melanoma, squamous head and neck, and non-small cell lung cancer patients combined with the negative immune checkpoint inhibitors pembrolizumab (anti-PD-1, NCT02575404, and NCT04987996, this last suspended) and ipilimumab (anti-CTLA-4, NCT02117362; **Table 2**). No results are available yet from those clinical studies. Interestingly, this compound has also been evaluated in clinical trials for non-alcoholic steatohepatitis, portal hypertension, and advanced liver fibrosis (NCT01899859 and NCT02462967, **Table 2**). In this case, GR-MD-02 was safe but not associated with significantly ameliorating hepatic disease (184).

An area under intense investigation tries to achieve formulations with improved pharmacokinetic properties for this type of carbohydrate-based inhibitors. This is the case of lactose-, galactose- or pectins-complexed nanoparticles (185–187). Apart from improving the pharmacokinetic properties of the inhibitor, these nanoparticles can also serve as delivery carriers of cytotoxic drugs toward the tumor (66, 188, 189). Moreover, attempts are being made with nanoparticle modifications to improve selective targeting of the tumor (or tumor-associated stroma) (67, 190).

Interestingly, non-carbohydrate inhibitors for galectins have also been proposed. First, the anti-tumor properties of several synthetic heterocyclic compounds able to bind galectin-1 have been evaluated. Molecular docking experiments described fine interactions between these molecules and the CRD domain of galectin-1 (191–194). Moreover, *in vitro* results indicate these compounds have anti-tumor cytotoxic properties (191–194). However, *in vivo* anti-tumor pre-clinical evaluations of such compounds remain to be performed. Second, bacteriophage display library systems for interaction screening allowed the discovery of galectin-binding peptides. For instance, a Thomsen-Friedenreich antigen-specific peptide (P-30) able to bind galectin-3 has been described (195). This peptide modulates breast and prostate tumor homotypic aggregation and tumor cell adhesion to the endothelium (195). Using similar technological approaches, stapled-peptides ligands binding galectin-3 were described (196). These peptides bind to the CRD of galectin-3 and the best one has an intermediate affinity (Kd 0.45 μM) (196). However, no functional studies have been reported for these peptides. As already mentioned, formulations with improved pharmacokinetics are being evaluated. In this context, nanoparticles combining carbohydrates (inhibitor) and peptides (addressers) have been described, a strategy that significantly improves their biodistribution and the biological effects (67).

Finally, genetic engineering methods are used to inhibit the glycan-dependent functions of galectins. For instance, a dominant negative mutant formed by the last 143 carboxyl-terminal amino acid residues and lacking the N-terminal domain of galectin-3 (named Gal-3C) has been described. This Gal-3C molecule preserves the CRD but lacks cooperative binding and crosslinking properties of the wild-type galectin-3 (197). Indeed, it is hypothesized that the administration of an excess of soluble Gal-3C competes with endogenous galectin-3 for carbohydrate binding sites (76). In this context, Gal-3C reduces angiogenesis by abrogating extracellular galectin-3 interaction with αvβ3 integrin through its carbohydrate recognition domain (198). Interestingly, Gal-3C inhibits CXCL12-induced leukocyte migration in (non-cancer) inflammatory conditions (199). Gal-3C also inhibits tumor cell motility and invasion (75, 200). Hence, Gal-3C alone or combined with other chemotherapies can reduce ovarian, breast cancer, and multiple myeloma growth and drug resistance (75, 76, 200). Interestingly, Gal-3C can be used *in vivo* without toxic effects (76); this treatment ameliorates heart failure after myocardial infarction (77). Galectin-9 mutants have also been described. Indeed, mutations in galectin-9 CRD abolish its binding to the negative checkpoint Tim-3; this interaction occurs *via* the carbohydrates (201). Dominant negative mutants can also interfere with nuclear partners in a glycan-dependent manner. This is the case of the interactions between galectin-1 and Foxp3. This transcription factor functions as a master controller of regulatory T cells (Treg). Moreover, the interaction between galectin-1 and Foxp3 controls a panoply of genes and functions in breast cancer cells (202). Consequently, galectin-1 mutants that lack the N-terminus and do not bind Foxp3 can be used to inhibit breast tumor proliferative and invasive properties (202). These results show that negative dominants could be interesting tools to inhibit galectins.

#### Non-competitive allosteric inhibitors of carbohydrate-binding to galectins

Some inhibitors do not directly interact with the CRD of galectins, but their inhibitory effects are still glycan-dependent. Indeed, these molecules function as allosteric inhibitors, interacting outside the CRD but inducing changes in this region, thereby inhibiting glycan binding and biological effects. For instance, *in vivo* galectin-1 inhibition through the administration of lactose-conjugated purpurinimide photosensitizers reduced the growth of radiation-induced fibrosarcoma (58). Molecular modeling analysis indicated that this compound does not interfere with the CRD (203). Similar photodynamic strategies with galactose-bound porphyrin demonstrated anti-tumor effects in bladder cancers (57). In this case, galectin-1 inhibition generates oxidative stress and apoptosis of tumor cells over-expressing this lectin (57).

However, allosteric inhibition can also be performed using non-carbohydrate molecules. Based on the significant role of galectins in the interaction between tumor and endothelial cells during tumorigenesis, a cytokine-like peptide named anginex was described as a potent anti-angiogenic tool (68). This biological effect is mediated through galectin-1 binding (69), although this peptide also binds other galectins (204). The anti-tumor effects of anginex were demonstrated in several experimental cancer models (26, 32, 43, 44). Anginex’s angiostatic beta-sheet-forming structure inspired the design of the 6DBF7, a peptidomimetic that also interacts with galectin-1 (70, 71). This 6DBF7 molecule inhibits glycan binding of galectin-1 in a noncompetitive, allosteric manner (71). Based on these studies, other potent analogs (DB16 and DB21) have also been described (71). These peptides inhibit angiogenesis and tumor growth significantly better than 6DBF7 or anginex (71). To overcome the susceptibility of these peptides to hydrolysis by proteases, Dings et al. designed a non-peptidic topomimetic of anginex and 6DBF7 based on a calixarene scaffold. Indeed, calix[4]arene compound 0118/OTX008/PTX008 binds to galectin-1 at a site away from the lectin’s carbohydrate binding site, thereby attenuating lactose binding to the lectin (205). It should be mentioned that the specificity of this compound is relative since it also binds to galectin-3, albeit more weakly (206). Pharmacokinetics and anti-tumor activity of OTX008 alone or combined with other treatments were evaluated in melanoma, glioblastoma, thyroid and ovarian carcinoma (40, 43, 72, 73, 207). A phase I study of OXT008 in patients with advanced solid tumors was reported (NCT01724320, **Table 2**). Unfortunately, this study is listed with an “unknown recruitment status”; no updates have been posted since 2012. Chemical modifications of PTX008 were also described; it is interesting to mention the PTX013 compound. This compound is more potent as a cytotoxic tumor agent than the parenteral PTX008. This higher inhibitory potency of PTX013 was demonstrated both *in vitro* (head and neck, breast, ovarian, renal, lung, and prostate cancer lines, several of them radiation resistant), and importantly *in vivo* (melanoma) (74).

Galectin inhibition can also be achieved using specific neutralizing monoclonal antibodies (mAb). It must be noted that, for this strategy, mechanisms of galectin inhibition (competition or allosteric inhibition) depends on each antibody. In the case of galectin-1, one of these antibodies (Gal-1-mAb3) has been characterized, and the epitope recognized by this mAb localizes outside the CRD although it is still capable of inhibiting N-acetyllactosamine-galectin-1 interaction (208). This antibody recognizes specifically galectin-1 with high affinity (EC50 = 523nM). This neutralizing antibody reproduces the anti-angiogenic and immunopotentiating activities observed with other types of inhibitors (208, 209). In particular, blockade of galectin-1 (Clone 25C1; Novo Castra) significantly reduces the *in vitro* inhibitory effects of human and mouse CD4+CD25+ Treg cells (210). Moreover, another anti-galectin-1 neutralizing mAb ameliorates the negative immune checkpoint (PD1) response in irradiated mice carrying oral cancer cells (45).

In the case of galectin-3, earlier studies described mAbs recognizing non-CRD domains but causing a profound modulation of its lectin activities (211). On the other hand, a galectin-3-specific mAb (14D11) competes with lactose for the carbohydrate-binding pocket of galectin-3 (81). This antibody inhibits invasion of Mucin-16-expressing cancer cells, prolonging overall survival in animal tumor models (81). However, inhibition of galectin-3 also impacts the tumor stroma cells. Indeed, the use of an anti-galectin-3 mAb (B2C10) promotes IFN-γ secretion by *in vitro* stimulated CD8+ tumor-infiltrating T lymphocytes (63).

The scientific interest in developing anti-galectin-9 mAb is major since this protein participates in various mechanisms of immune escape by tumors: control of T cell survival (212), T cell effector exhaustion and differentiation (82, 201, 213, 214), lymphocyte migration towards the tumor *via* an endothelial cell reprogramming (45), Treg function (215–220), regulation of antigen presentation (221–223), and myeloid suppressive cells (224). Confirmation of these functions by the use of blocking antibodies is becoming very frequent. Such is the case of two antibodies (clones 292-13 and 292-18A) reacting with high affinity with the N-CRD of human galectin-9; their use protects T cells from galectin-9 mediated cell death and promotes tumor-cell killing by T cells (225). The same group, but using a commercial anti-galectin-9 mAb (RG9-1 from InVivoMAb), demonstrated prevention of CD8+T cell exhaustion and near complete Treg depletion when this mAb is combined with anti-GITR (glucocorticoid-induced tumor necrosis factor receptor-related protein)-specific antibody (82). Two other anti-galectin-9 mAb have also been reported (Gal-Nab1 and Gal-Nab2). In this case, antibodies recognize an epitope comprising 213-224 amino-acid sequence with high affinity (in the order of nM) (226). Again, these antibodies protect T cells from galectin-9-mediated cell death (226). An anti-galectin-9 was combined with anti-Tim-3 mAb to improve taxane-based chemotherapy in breast cancer (83). Apart from the direct effects on adaptive immunity, blockade of galectin-9 by antibodies potentiates immune attack in pancreatic carcinoma through modulation of macrophage function (84). Nevertheless, galectin-9 blockade by antibodies also acts directly on tumor cells. Indeed, leukemia stem cells secrete galectin-9, which through the interaction with Tim-3 constitutes an autocrine loop critical for leukemic self-renewal and development (85). Indeed, galectin-9 neutralization is a potent way to prevent the reconstitution and the self-renewal of human acute myeloid leukemia cells in a xenogeneic transplantation model (85). Finally, an anti-galectin-9 mAb (Lyt-200) is currently under clinical investigation in phase I/II trial for its safety and efficacy in patients with relapsed/refractory metastatic solid tumors (NCT04666688, **Table 2**). In this clinical trial, Lyt-200 is evaluated alone and in combination with chemotherapy or anti-PD-1.

The use of neutralizing antibodies to block other galectin members in cancer is more incipient, and in most cases, polyclonal antibodies are evaluated. For instance, neutralizing surface-bound galectin-4 in human colorectal cancer induces significant transcriptional changes and chemokines production in tumor cells (227).

While neutralizing antibodies carry several benefits over small inhibitory carbohydrate molecules, they also have several drawbacks. Some of the concerns are related to their selectivities and biodistributions. Antibodies inhibit extra-cellular galectins, and lack restricted biodistribution in the body. These characteristics imply that antibody-mediated inhibition of galectins could act as partial inhibitors (lack of intracellular effects), and do not discriminate between non-transformed and transformed cells resulting in adverse effects. More studies are needed to fully understand the effects induced by galectin-neutralizing antibodies and their potential transfer to the clinic.

Finally, nucleotide-based molecules are a different family of galectin inhibitors. In this sense, a single-stranded DNA aptamer (AP-74 M-545) has been described as an antagonist of galectin-1 (86). This aptamer shows higher affinity (KD = 3.7 nM) and specificity than the previous inhibitors. Administration of this compound induces *in vivo* anti-tumor effects through activation of the immune system. Indeed, this aptamer prevents T cells from apoptosis and restores T cell-mediated immunity (86). This study did not evaluate aptamer dependence on glycans, so this point remains to be clarified.

### Carbohydrate-independent galectin inhibitors

Apart from their extracellular glycan-dependent functions, galectins also display intracellular functions, most of which are glycan-independent. Therefore, the development of molecules inhibiting these functions may be convenient. In this respect, small benzimidazole compounds (LLS2 and the improved LLS30) bind to the interface between the dimeric galectin-1 subunits within 6 Å from the β-galactoside binding pocket (106). The binding of these compounds to galectin-1 decreased membrane-associated H-Ras and K-Ras and contributed to the suppression of CXCR4, pErk, and AKT signaling pathways (88, 106, 107). Interestingly, pre-treatment of prostate tumor cells with LLS30 reduced their adhesion on collagen-, fibronectin-, and laminin-coated surfaces (107). *In vivo* administration of these compounds promotes anti-cancer effects in ovarian (106), hepatic (87), malignant peripheral nerve sheath (88), and prostate (107) pre-clinical cancer models. Importantly, combining these compounds with taxanes in *in vitro* and *in vivo* experiments resulted in synergistic cytotoxicity against several human cancer cell lines (ovarian, pancreatic, prostatic, and breast cancer cells) (106). These compounds have a direct cytotoxic effect on tumor cells and the cancer-associated stroma (*e.g.*, fibroblasts) (87).

In addition, two tetrahydroisoquinoline natural products (DX-52-1 and HUK-921) inhibit cell migration through interactions with galectin-3 (228). This interaction occurs outside the β-galactoside-binding site of galectin-3. While this compound’s exact mechanism of action remains to be understood, experiments demonstrated that this effect is glycan-independent (228).

While the use of dominant negative mutants for *in vivo* therapies is still way off, this type of inhibitor allowed us to understand several aspects of the glycan-independent intracellular signaling of galectins. For example, galectins-1 and -3 are constituents of the pre-mRNA splicing machinery (229–233). This interaction is glycan-independent (234), and a N-terminal galectin-3 polypeptide exhibited a dominant negative effect on splicing (231). Interestingly, silencing of galectin-3 was sufficient to alter the splicing patterns of several genes, including the transcripts coding for the SET nuclear oncogene (235). Moreover, galectin-3 regulates promoter activity of different genes highly involved in malignant transformation such as cyclin D1 (236), FOXD1 (237), the thyroid-specific transcription factor TTF-1 (238), and MUC2 (239). A galectin-3 mutant that cannot be phosphorylated at the Ser6 site demonstrated that this post-translational modification is critical for galectin-3 function as a modulator of gene expression (78, 240).

At the cytoplasm, galectins-1 and -3 are recruited by the small GTPase Ras, which become integral parts of plasma membrane nanoclusters (241). Indeed, mutations in a hydrophobic pocket of the galectin-1 CRD induce a dominant negative mutant that cannot interact with H-Ras anymore and, therefore, abrogates signal output (242). Nevertheless, the biological interaction between galectins and Ras does not depend on carbohydrate binding (242, 243). Inspired by that observation, a galectin-3 dominant negative was also created. Similarly, this galectin-3 dominant negative does not interact with K-Ras anymore and abrogates signal output from the Raf/mitogen-activated protein (MAP)/extracellular signal-regulated kinase (ERK; MEK) pathway (241, 244, 245). This initial molecular model of galectin-Ras interactions was then revised by demonstrating that galectin-1 does not directly bind to H-Ras, but instead to the Ras binding domain of Ras effectors, such as Raf (246). Whatever the exact interactor in Ras signaling, galectin-1 and -3 dominant negative mutants reduce cell growth and transformation (243–245). Finally, dominant negative galectins interfere with another type of cytoplasmic interactions with regulatory potential for tumorigenesis. Indeed, galectin-3 bears the NWGR conserved motif with several members of the Bcl-2 family, and using a galectin-3 mutant modifies this delicate balance between cell survival and death (247). In conclusion, several reports have shown the utility of inhibiting the carbohydrate-independent functions of galectins. No report is yet found on their use in pre-clinical as well as clinical trials.

### Negative control of galectin gene expression (ablation of all its functions)

Since the description and widespread use of RNA interference to control gene expression, its use to inhibit galectins has been intensive. RNA interference strategies include transient (siRNA) or stable (shRNA-encoding vectors) effectors. Interestingly, this strategy should affect galectin functions more than former inhibitors since it modulates glycan-mediated and -independent effects, and with higher specificity since the nucleotide sequence is highly different between galectins’ members. It is impossible to cite all the publications that have used this approach to downregulate galectins in this review; we only mention a few examples. Indeed, RNA interference was often used to confirm basic aspects of tumor biology (which includes intrinsic effects on the transformed cells themselves (88–91, 99–101, 103, 237, 248–258), the modulation of the tumor-associated stroma (80, 96, 97, 201, 259–265) and, importantly, as a synergic therapy option for cancer (37, 42, 92, 98, 101, 102, 104, 266–270). Several properties of this gene control strategy deserve to be highlighted compared to the aforementioned galectin inhibitors. First, these inhibitory molecules have the highest reported affinities for their messenger RNA target. Indeed, siRNA concentrations in the picomolar range can induce efficient gene expression knockdown, and intracellular amounts of less than 2,000 siRNAs molecules per cell were demonstrated to induce potent biological effects (271). Second, the actions of this type of inhibitor are highly specific. Indeed, siRNAs can downregulate the expression of mRNA transcripts through a highly specific nucleotide hybridization process; it can differentiate single base changes in genes (272, 273). These two properties (affinity and selectivity) make siRNA (and their chemical modifications) an efficient approach to inhibit any target through their gene expression knockdown, and their evaluation in clinical trials is promising [reviewed in (274–277)]. Although protein-based drugs, including monoclonal antibodies, are highly specific, their targets are primarily limited to cell surface receptors or circulating proteins. On the contrary, specific degradation of the galectin transcript by siRNA leads to significant protein downregulation, affecting all the functions galectins are involved in, independently of their glycan dependence. However, various hurdles must be resolved before bringing siRNA into clinical use. First, a selective biodistribution (it would be highly desirable to address siRNA towards the tumor or the tumor-associated stroma, avoiding a non-specific biodistribution that would be responsible for adverse effects). Second, it is needed to improve siRNA stability and reduce their clearance to increase their half-life in the biological fluids. Finally, it is necessary to prevent off-target effects including nucleotide-based immune activation (278, 279). To do this, delivery systems have been developed to protect siRNA from nuclease degradation and facilitate cellular uptake at target sites [chemically modified RNAs (280, 281), nanoparticles (37, 92, 93, 282) and lipoplexes (283)]. These strategies have demonstrated effectiveness to some extent. However, all these approaches face different problems concerning safety, production costs, and often poor correlation between *in vitro* and *in vivo* efficacy, making their development a significant challenge.

On the other hand, the endogenous expression of several galectins is subject to gene control by miRNAs. It has been reported that miRNA-22 and -2467 regulate the expression of galectin-1 (284–286), miR-424-3p, -873 and -128 regulate galectin-3 (105, 287–290), miR-1236-3p regulates galectin-8 (291) and miR -455-5p and -22 regulate galectin-9 (292, 293). This finding offers another level of intervention that could be of great interest as therapeutical strategies for various cancers. For example, the utility of miR-424-3p modulation has been demonstrated for ovarian and colorectal cancers (105, 287, 288). In this regard, it has been shown that resveratrol stimulates the transcription of miR-424-3p, which suppresses the expression of galectin-3 (105). In the future, it is expected that the development of gene control strategies through miRNAs will provide new means for controlling galectin levels in the tumor microenvironment.

Finally, developing genome editing strategies such as CRISPR Cas-9 for galectins in the clinic is confronted with ethical obstacles (induction of genome alterations in non-targeted cells) (294, 295). Indeed, the safe and effective delivery of genome editing enzymes represents a substantial challenge that must be tackled to enable the next generation of genetic therapies. However, such genetic strategies will probably contribute to a better fundamental understanding of the role of galectins in cancer. Despite this limitation regarding their direct *in vivo* use in cancer patients, these strategies could represent real options for *in vitro* approaches (development of cell-based anti-tumor vaccines or cell conditioning before being infused into patients) (296).

## Challenges for clinical application of galectin inhibitors

This chapter itemizes the properties that differentiate galectin inhibitors from each other, and that should be taken into account when scaling up their use in the clinic:

*1-Affinity*: this is one of the most distinctive parameters of current inhibitors. In general, molecules with higher affinity will require lower doses to obtain *in vivo* biological effects and, therefore, may induce fewer adverse effects (297, 298). However, it should be noted that affinity calculations are performed *in vitro*; these molecules have IC50 (inhibitory concentration 50) ranging from μM to pM (as discussed throughout the review for each type of inhibitor). Routine methods used to measure affinity and selectivity include fluorescence polarization binding (299), competitive binding enzyme-linked immunosorbent assays (300), isothermal titration calorimetry (301), biolayer interferometry (138), and surface plasmon resonance (302). These binding assays primarily focus on the CRD, although other non-CRD interactions can also be detected (159). In the case of genetic-based strategies, inhibitors are evaluated by determining galectin transcript or protein levels and functional assays. This methodological heterogeneity makes assessing inhibition potency a real challenge. In addition, although these *in vitro* determinations allow the compounds to be compared with each other in controlled conditions, they do not define their real inhibitory capacity *in vivo*. Indeed, in addition to affinity determination in controlled conditions, several other parameters will determine their *in vivo* inhibitory potential. We can cite their abilities to diffuse across membranes (which determine their tissue biodistribution and extra/intra-cellular localization), the properties of the local microenvironment, and the presence of other biological competitive interactors (141).
*2-Specificity for a galectin member (and isoform)*: this is another fundamental challenge in the field of galectin inhibitors due to the high amino acid sequence homology in the core site between the different members of the galectins (303, 304). Compounds should recognize the correct galectin member. Moreover, several galectin members display multiple isoforms generated from alternative splicing [we can cite galectins-8 (305), -9 (306), and -12 ((307, 308) *LGALS12* galectin 12 [Homo sapiens (human)]-Gene-NCBI)]. In this context, gene inhibition strategies are compelling alternatives in terms of specificity. However, other post-translational modifications generate galectin variants such as the cleaved or phosphorylated forms of galectin-3 (240, 309–311) and the O-GlcNAcylation of galectins; this last modification plays a major role in their secretion (312–315). Furthermore, it is worth noting that the quaternary structural conformations of galectins are highly dependent on the properties of the microenvironment. For example, the balance between galectin-1 monomers and dimers depends on the redox state of the cellular microenvironment (316).
Inhibitor specificity is a major point since different galectin members (and even different isoforms) often induce opposite biological effects (317–319) (240, 311, 320, 321). Therefore, the *in vivo* biological results can be complex if compounds simultaneously inhibit different galectin members (or different isoforms). Furthermore, many galectins play relevant physiological roles (13, 322). Thus, the ideal galectin inhibitor should alter tumor pathology without affecting physiological processes. These inhibitory molecules should be as selective as possible for a particular galectin member (appropriate isoform).
With state-of-the-art, it is not easy to establish a pecking order as to which galectin member should be inhibited to obtain maximal anti-cancer effects. All scientific reports that focus on individual galectins extol their experimental findings. However, to our best knowledge, no systematic study compared the anti-cancer effects obtained by inhibiting multiple galectins (individually or combined) using the same experimental design, especially considering the *in vivo* complexity. In addition, this scenario is complex since each type of cancer has particularities, so this study must be carried out for each cancer.
*3-Galectin function(s) that should be inhibited in cancer:* galectin-mediated biological processes in cancer involve interactions more complex than initially proposed and not only restricted to glycan-dependent ones (**Figure 1**). In this context, there is a lot of information about the glycan-dependent functions of galectins. On the contrary, our comprehension of the glycan-independent ones is more limited. At this level, an implicit question in selecting the best galectin inhibitory strategy for cancer is: what function(s) of these proteins should be preferentially inhibited? Is it sufficient to inhibit the lectin-mediated functions of galectins, or should the non-lectin functions also be inhibited for maximum anti-tumor activity? Noteworthy, complete inhibition of galectins by RNA interference-based approaches was generally used to confirm already-known biological functions of galectins (**Table 1**). To the best of our knowledge, no new biological functions have been reported by using these approaches. Therefore, more research is needed to clarify this point and to define which galectin functions should be targeted for cancer treatments.
*4-Where galectin inhibition should be accomplished:* This point is closely related to the previous one. Since galectins play relevant physiological functions, it would be highly advantageous to inhibit them selectively where they play a role in tumorigenesis. In this sense, we have some clues for certain galectins. For instance, galectin-1 downregulation in transformed (17, 123, 131–135) and tumor-associated stroma cells (46, 47, 125, 126, 136) have demonstrated beneficial effects in pre-clinical studies. Therefore, these reports clarify the cellular targets where galectin-1 should be inhibited to obtain beneficial anti-tumor effects. In addition, the sub-cellular localization where galectins play their functional roles must also be considered. For instance, galectin-3 was described at different sub-cellular compartments; inhibition of this protein in each of these localizations often causes opposite biological effects (182). These questions should be addressed for all the galectin members.
*5- Appropriate pharmacokinetics; specific biodistribution towards the cell targets:* Several of these inhibitors are polar molecules, of low molecular weight, with different capabilities to diffuse through the plasma membrane and, therefore, acting inside the cell (141). On the contrary, large molecules such as inhibitory antibodies are predicted only to engage extracellular galectins. Moreover, like most molecules, galectin inhibitors are trapped in organs with high blood flow, such as the liver, and inactivated through metabolic processes. Moreover, small molecules generally suffer rapid renal clearance (323). Such phenomena reduces the half-life of these molecules, and in consequence, their inhibitory efficiency. Furthermore, other pharmacokinetic properties may also be taken into consideration. In particular, many of the inhibitors are sensitive to enzymatic hydrolysis by glycosidases (324), proteases (325) or nucleases (326). Additionally, inhibitors’ random biodistribution can generate adverse effects due to the inhibition of galectins in tumor-unrelated cells. Therefore, developing degradation-resistant molecules with tumor (and its stroma)-selective biodistribution would be highly desirable.
*6- Not expensive and easy translation to clinics* should also be addressed.
7- *Development of resistance to inhibitory treatments:* tumors are highly dynamic biological entities capable of surviving by inducing resistance mechanisms. In the case of inhibiting the lectin functions of galectins, it is worth noting that the glycome is highly adjustable (by enzymatic remodeling without requiring neosynthesis). Thus, we might think that tumor cells would be capable of changing the glycan structures through sialylations (327) or sulfations (328); modifications which have a high impact on galectin biological effects. Otherwise, the same reasoning applies to glycan-independent functions of galectins and resistance development. In this context, it has been shown that the synergism between different treatments allows the use of lower doses of compounds and thus avoids the development of resistance (329). Therefore, this topic represents a significant issue for their transfer to the clinics.

Faced with the critical challenges of galectin inhibitors, regulating the cell glycosylation pattern appears as an alternative option [reviewed in (330, 331)]. Indeed, the creation of glycan ligands for galectins depends on the activities of various glycosyltransferases and glycosidases in the cell (332). In pre-clinical studies, glycome regulation is obtained through control of glycosyltransferases and glycosidases-coding genes (333–339), the use of metabolic inhibitors of glycan biosynthesis (340, 341), or carbohydrate-specific and blocking antibodies (342–344). While such biological disruptions are easily obtained at a pre-clinical level, their therapeutic implementation in patients must also overcome important challenges. In particular, as the glycome is a major determinant of multiple physiological processes, it is essential to avoid side effects. Once again, this type of intervention should be tumor (or tumor-associated stroma)-selective. Moreover, it is pertinent to point out that glycome regulation would only affect some galectin functions (those glycan-dependent). On the other hand, certain galectin inhibitors affect broader functions (including glycan-independent ones such as gene control). The authors consider that both strategies (galectin and glycome regulations) should be evaluated more in-depth, and synergistic or additive anti-tumor effects could be obtained through their combinations.

## Final considerations

The first reports about the usefulness of galectin inhibitors appeared in the early 2000s. Since then, a remarkable compendium of basic studies supports their potential utility in cancer, especially in synergy with other treatments (**Table 1**). However, none of the described galectin inhibitors have achieved clinical success; most did not go beyond the initial phases of clinical trials (**Table 2**). A detailed analysis of this **Table 2** shows that most studies did not translate into better treatments for patients, not even in a better fundamental understanding, as results are often not reported. Therefore, clinical and pre-clinical results must be communicated (even if the observed results differ from those expected) since they contribute to the continuous amelioration of these strategies.

Analyzing all the inhibition strategies reported so far, the authors opine that molecular biology techniques (*e.g.*, RNA interference) offer attractive advantages in affinity and member specificity compared to inhibitors with a carbohydrate nature or those obtained from chemical synthesis. In the case of blocking antibodies, there are important biodistribution drawbacks, which limit galectin inhibition in specific cellular compartments. Despite these particular aspects, much remains to be understood about the pharmacokinetic parameters, toxicity, and tumor resistance mechanisms for all galectin inhibitors.

Finally, since the available literature indicates that galectin inhibition induces effective anti-tumor effects, especially when combined with other strategies (e.g., irradiation, anti-angiogenic, chemotherapies, etc.), this concept should also be considered when designing therapeutic approaches. We conclude that many basic studies are still needed for an efficient clinical translation of galectin inhibitors.

## Author contributions

DL and DC writing—review and editing. All authors contributed to the article and approved the submitted version.
